# Multifunctional cytokine production reveals functional superiority of memory CD4 T cells

**DOI:** 10.1002/eji.201848026

**Published:** 2019-06-13

**Authors:** Lotus M Westerhof, Kris McGuire, Lindsay MacLellan, Ashley Flynn, Joshua I Gray, Matthew Thomas, Carl S Goodyear, Megan KL MacLeod

**Affiliations:** ^1^ Centre for Immunobiology Institute of Infection Immunity and Inflammation, 120 University Place University of Glasgow Glasgow UK; ^2^ GLAZgo Discovery Centre Institute of Infection Immunity and Inflammation University of Glasgow Glasgow UK; ^3^ Respiratory, Inflammation and Autoimmunity IMED AstraZeneca Gothenburg Sweden

**Keywords:** Cytokine, Influenza virus, Memory cell, Multifunctional, T cell

## Abstract

T cell protective immunity is associated with multifunctional memory cells that produce several different cytokines. Currently, our understanding of when and how these cells are generated is limited. We have used an influenza virus mouse infection model to investigate whether the cytokine profile of memory T cells is reflective of primary responding cells or skewed toward a distinct profile. We found that, in comparison to primary cells, memory T cells tended to make multiple cytokines simultaneously. Analysis of the timings of release of cytokine by influenza virus‐specific T cells, demonstrated that primary responding CD4 T cells from lymphoid organs were unable to produce a sustained cytokine response. In contrast CD8 T cells, memory CD4 T cells, and primary responding CD4 T cells from the lung produced a sustained cytokine response throughout the restimulation period. Moreover, memory CD4 T cells were more resistant than primary responding CD4 T cells to inhibitors that suppress T cell receptor signaling. Together, these data suggest that memory CD4 T cells display superior cytokine responses compared to primary responding cells. These data are key to our ability to identify the cues that drive the generation of protective memory CD4 T cells following infection.

## Graphical abstract

Influenza virus‐specific memory CD4 T cells are more likely to be multifunctional and produce a more sustained cytokine response than primary responding T cells.

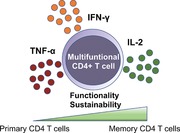

## Introduction

Immunological memory provides superior immune protection from pathogens for two reasons. First, there are more pathogen‐specific cells present in the memory as compared to the naïve compartment. Second, memory cells are intrinsically different from naïve cells: they are located in peripheral organs as well as in secondary lymphoid organs; are more sensitive to activation signals; and provide a more tailored response 1, 2.

This tailored response is defined by the effector cytokines T cells express and is one of the main mechanisms of T cell‐mediated immune protection. The ability to track antigen‐specific T cells via their cytokine profile is essential to understand their protective potential. This cytokine profile is shaped by pathogen‐triggered signals during the primary response 3. These signals drive the effector cytokine production, such as IFN‐γ, IL‐4, or IL‐17, most effective at coordinating pathogen control 2, 4.

Multiple studies demonstrate that memory T cells can develop from differentiated, effector cytokine producing cells 5, 6, 7, 8. However, there is also evidence that the least differentiated cells are more likely to enter the memory pool suggesting that these cells may dominate the memory pool 9, 10, 11. Such cells may not, however, offer the best protection. The most effective memory T cells are thought to be cells that produce high levels of a range of different cytokines, termed poly or multifunctional T cells 12, 13, 14, 15. While it is not yet clear why multifunctional memory T cells offer the most effective protection, their ability to produce cytokines, such as IFN‐γ, that activate innate immune cells and IL‐2, which aids T‐cell proliferation, means they both drive and sustain secondary responses.

Understanding the relationship between primary responding and memory T cells will aid in the design of vaccines that aim to drive protective immunological memory. A major hurdle in characterizing pathogen‐specific T cells is in their identification. Most studies use either monoclonal TCR transgenic CD4 T cells or identify endogenous T cells specific to a single epitope 16, 17, 18, 19, 20. This narrow focus limits the research scope, especially as CD4 T cells are thought to respond to a diverse range of pathogen epitopes 21, 22.

Here, we address how the cytokine profile of endogenous polyclonal pathogen‐specific effector CD4 and CD8 T cells relates to that of subsequently generated memory T cells. We find that, in comparison to primary effector T cells, memory CD4 T cells have an increased tendency to be multifunctional, display a more sustained cytokine response, and are less sensitive to inhibitors of TCR signaling. Memory CD8 T cells are also more likely to be multifunctional than primary responding cells. However, primary and memory CD8 T cells are similar in the sustainability of the cytokine response and their sensitivity to TCR signaling inhibitors.

## Results

### Cytokine producing CD4 T cells decline most dramatically in the lung

The primary immune response is usually followed by a contraction phase in which most activated T cells undergo cell death 23. Highly differentiated effector cytokine producing T cells are thought to be more likely to undergo apoptosis than less differentiated IL‐2‐producing cells 18. Moreover, memory cells in peripheral tissue are thought to be more differentiated than those in lymphoid organs and the bone marrow has been proposed as a site of memory T cell maintenance 24, 25, 26, 27, 28, 29.

To address these assumptions, we examined the ex vivo cytokine responses of CD4 and CD8 T cells isolated from mice infected with influenza A virus (IAV) at the peak of the primary responses, day 9, and at two memory time points, days 30 and 75. We identified IAV‐specific T cells by their ability to produce cytokine, following ex vivo restimulation with bone marrow‐derived dendritic cells (bmDCs) incubated overnight with IAV. Incubation of bmDCs with IAV (IAV+ bmDCs) caused minor upregulation of MHC II and costimulatory molecules (Supporting Information Fig. 1).

**Figure 1:**
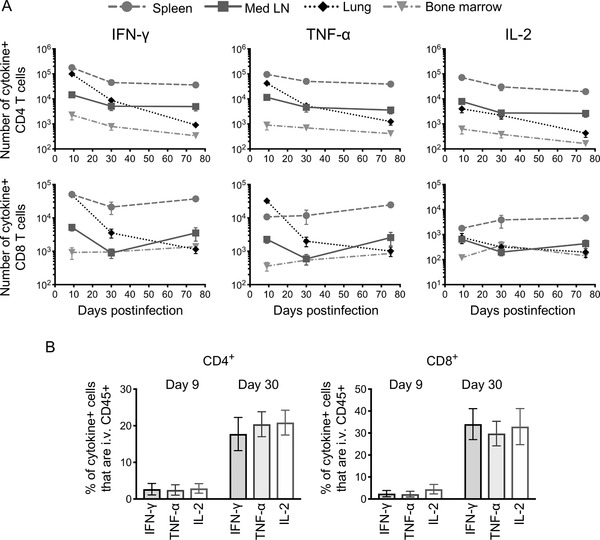
Cytokine producing IAV‐specific memory CD4 and CD8 T cell numbers stabilize in lymphoid organs but continue to decline in the lung. C57BL/6 mice were infected with IAV and 9, 30, or 75 days later the IAV‐specific cytokine+ T cells were examined by flow cytometry following 6 h restimulation with IAV+ bmDCs. Mice were injected with fluorescently labelled anti‐CD45 shortly before tissues were harvested. In A, each point represents the mean of 8–9 mice combined from two independent experiments; error bars are SEM and numbers are the absolute numbers of indicated cell types present in each organ. In B, the percentages of cytokine+ CD4 and CD8 T cells that bound to the injected anti‐CD45+ and were IFN‐γ+ at days 9 and 30 are shown; each point represents one mouse and the line shows the mean of the group. Data are combined from three experiments with 3–4 mice per group.

In all organs, we identified populations of IFN‐γ, TNF‐α, and IL‐2 IAV‐specific T cells (Supporting Information Figs. 2 and 3). Overall, the number of cytokine+ IAV‐specific CD4 T cells declined from the peak response at day 9 and then numbers levelled out (Fig. 1A). The decline in numbers of cytokine producing CD4 T cells was most obvious in the lung for IFN‐γ+ cells and least obvious in the bone marrow for TNF‐α and IL‐2+ cells, although this organ contained the smallest numbers of cytokine+ cells. Similarly, IAV‐specific CD8 T cells declined from day 9 to day 30, although the small number of IL‐2+ cells remained fairly constant. After the contraction phase, the numbers of cytokine+ CD8 T cells largely remained steady. For CD4 T cells, the memory cells were predominantly found in the spleen and mediastinal lymph node, while CD8 cytokine+ T cells were mainly found in the spleen.

The dramatic decrease of T cells in the lung could have been due to a large population of IAV‐specific T cells within the lung vasculature at day 9. However, by labelling these cells in vivo with fluorescently labelled anti‐CD45 shortly before the tissues were harvested 30, we found that more IAV‐specific cytokine+ T cells were found in the blood at memory as compared to primary time points (Fig. 1B).

Together these data confirm that, as expected, cytokine+ T cells contract from the peak of disease. However, our data suggest that cytokine+ memory T cells in lymphoid organs are just as likely as those from peripheral tissues to make effector cytokines. We did not observe preferential survival of memory CD4 or CD8 T cells in the bone marrow and, given the small numbers of memory CD4 T cells present in the bone marrow did not pursue these cells further.

### Memory T cells are more likely to be multifunctional than primary responding T cells

Given that IL‐2, particularly autocrine IL‐2, is thought to support memory T‐cell development 18, we expected to see an increased proportion of IL‐2 producing T cells in the memory pool. We did not observe a dramatic shift toward IL‐2+ IAV‐specific CD4 T cells within the memory pool (Fig. 1). Rather, similar numbers of IAV‐specific cells identified by any one of the three cytokines suggested that these may be the same cells that produce all three cytokines. To address this, we examined the combined cytokine producing capacity of individual cells in both the primary and memory response.

**Figure 2:**
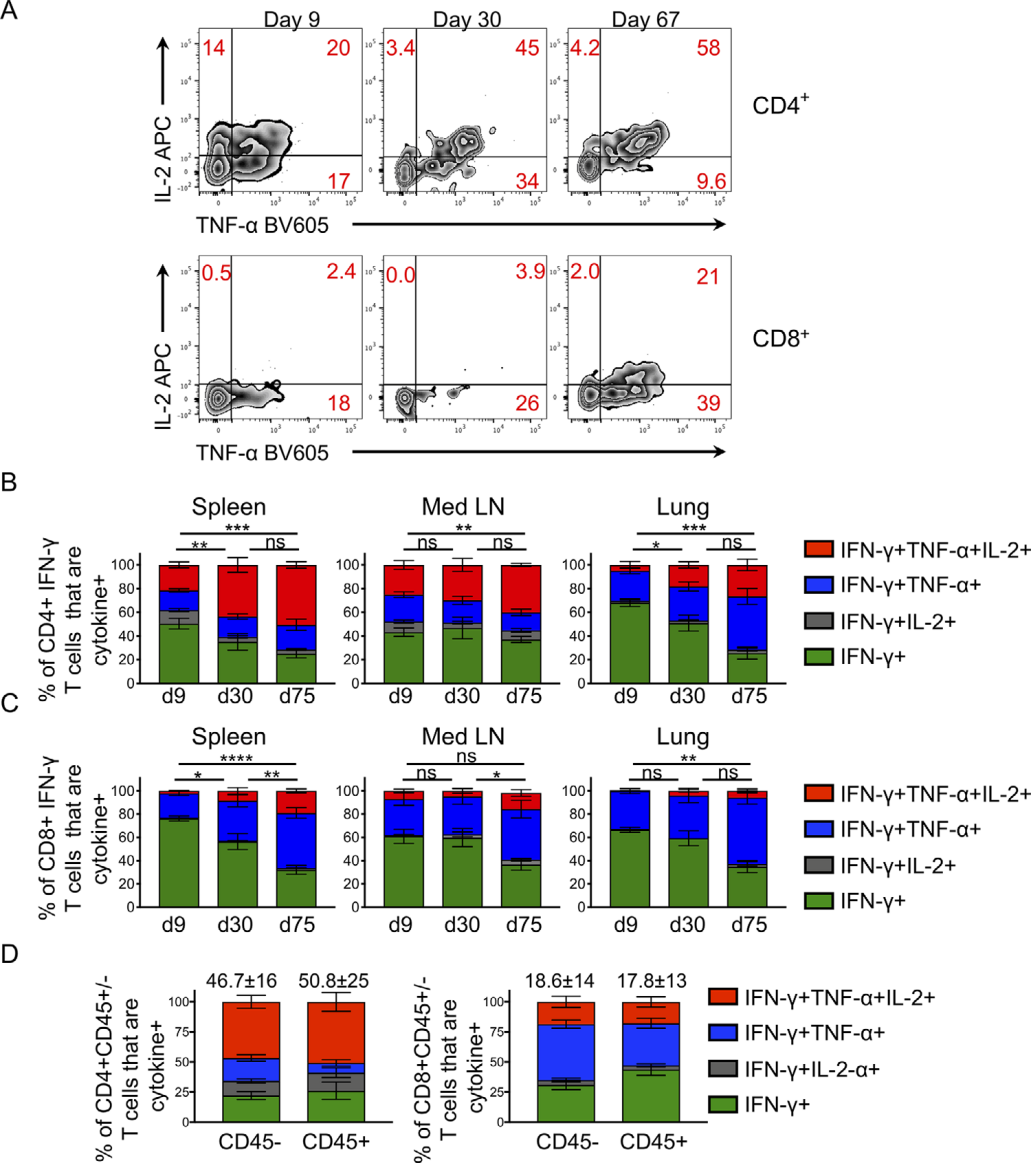
Multifunctional CD4 and CD8 T cells increase in proportion from the primary to the memory pool. Mediastinal lymph nodes, spleens, and lungs were taken from C57BL/6 mice 9, 30, and 75 days postinfection with IAV. The percentages of IFN‐γ+ CD4 and CD8 T cells that also expressed IL‐2 and/or TNF‐α were determined following 6 h stimulation with IAV+ bmDCs. In A, spleen cells are gated on live CD4+ or CD8+ dump negative lymphocytes that are IFN‐γ+; representative FACS plots from three experiments with three to five mice per group. In B and C data are from two time course experiment with a total of eight to nine mice/group/time point. In D, mice infected with IAV 30 days previously were injected i.v. with fluorescently labeled anti‐CD45 3 min prior to euthanasia and lung cells stimulated and examined as in A and numbers show mean + SD of the triple+ population. Data in D are from three experiments with a total of 10 mice. Samples were analyzed by ANOVA followed by a Tukey's multiple comparison test. *p < 0.05; **p < 0.01; ***p < 0.001; ****p < 0.0001.

For CD4 T cells, we observed a consistent increase in the proportion of multifunctional cytokine+ cells that produced IFN‐γ, TNF‐α, and IL‐2 in all three organs examined (Fig. 2). This shift was associated with a reduction in single IFN‐γ+ cells. At day 9, minimal IL‐2 was made by CD8 T cells. However, memory CD8 T cells are more likely to make IL‐2 than primary responding cells 11, 31, 32. We confirmed this and found that, as with the CD4 T cell response, the memory CD8 T cell pool was more likely to contain multifunctional CD8 T cells.

The increase in multifunctional T cells in the memory pool observed in the lung could have been due to the increased proportion of cells in circulation rather than a shift in multifunctionality in cells within the lung itself. However, for both CD4 and CD8 T cells we found similar proportions of multifunctional cells within the i.v. injected CD45 positive and negative fractions 30 days postinfection. This demonstrates that circulating and tissue resistant populations displayed similar multifunctional characteristics and support the idea that memory cells, regardless of location, shift toward increased multifunctionality (Fig. 2D and Supporting Information Fig. 4).

### The dynamics of T‐cell cytokine release reveals functional maturation of CD4, but not of CD8, T cells

To investigate the dynamics of cytokine production by T cells, we analyzed the kinetics of cytokine secretion during the ex vivo restimulation. T cells from mice infected with IAV 9 or 30 days previously were activated with IAV+ bmDCs, and Golgi Plug added at the start of the incubation, 2 or 4 h later. Cells were analyzed 2 h after the addition of Golgi Plug.

**Figure 3:**
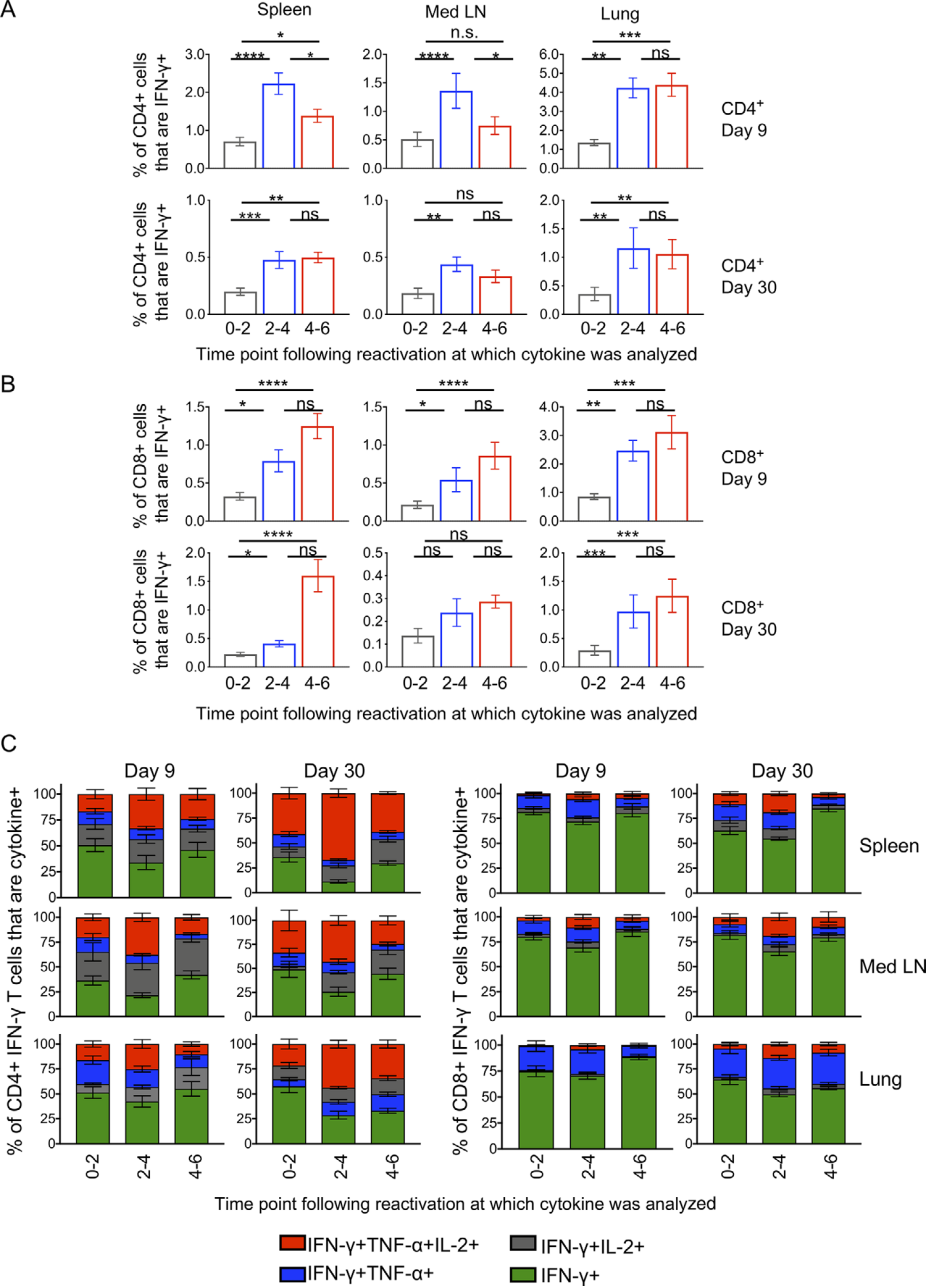
Memory CD4 T cells demonstrate more sustained cytokine production than primary responding cells. Mediastinal lymph nodes, spleens, and lungs were taken from C57BL/6 mice 9 and 30 days postinfection with IAV and reactivated in vitro with IAV+ bmDCs for 2, 4, or 6 h in the presence of Golgi plug for the last 2 h of culture. The percentages of IFN‐γ+ CD4 (A) and CD8 T cells (B) or IFN‐γ+ CD4 and CD8 T cells that also expressed IL‐2 and/or TNF‐α (C) were examined at the indicated time points. Error bars show SEM. Data are combined from three experiments per time point with four mice per time point. Samples were analyzed using a Friedman's paired test followed by a Dunn's multiple comparison test. *p < 0.05; **p < 0.01; ***p < 0.001; ****p < 0.0001.

Primary responding and memory CD8 T cells produced sustained or increased levels of cytokine, most notably IFN‐γ, throughout the restimulation period. However, primary responding CD4 T cells from lymphoid organs produced less cytokine at 4–6 h than at 2–4 h poststimulation (Fig. 3: IFN‐γ and Supporting Information Fig. 5: TNF‐α and IL‐2). These data suggest that antiviral CD4 T cells have a less sustained cytokine response than CD8 T cells.

In contrast, lung primary responding CD4 T cells produced sustained levels of cytokine suggesting that CD4 T cells from peripheral organs have altered regulation of their cytokine responses compared to those from lymphoid organs (Fig. 3 and Supporting Information Fig. 5). Interestingly, memory CD4 T cells, regardless of their source, displayed sustained cytokine responses with similar percentages of cells producing cytokine throughout the restimulation culture. These data suggest that memory CD4 T cells are functionally more superior than primary responding CD4 T cells.

An alternative explanation for this observation is that the primary responding pool contains a subset of T cells that only produce cytokine between 2 and 4 h following restimulation but that this subset is missing from the memory pool. To investigate this, we calculated the proportion of the number of CD4 T cells producing IFN‐γ at 2–4 h out of the number of IFN‐γ+ CD4 T cells found throughout the whole restimulation period (i.e. Golgi plug present from 0 to 6 h). These percentages were similar between the primary and memory CD4 T cells, regardless of which organ was examined (Supporting Information Fig. 6). These data suggest that there are similar proportions of the whole population responding at 2–4 h in the primary and memory CD4 T cells.

By examining cytokine production across the restimulation culture, we were able to determine whether triple, double, and single cytokine producing T cells had similar kinetics of cytokine production. This was the case for CD4 T cells, with all populations present at the three time points examined (Fig. 3C). A similar pattern was found for CD8 T cells, although the percentages of multifunctional cells in the primary response were very low. These data show that multifunctional cells are indeed able to produce all three cytokines simultaneously and that these cells have similar kinetics of cytokine release as double and single cytokine producers at primary and memory time points.

### PI3kinase inhibitors reveal altered cytokine responses by primary responding and memory CD4 T cells

The different cytokine profiles of IAV‐specific CD4 T cells between organs and time points prompted us to investigate whether cytokine production was regulated distinctly in these cells. PI3Kinases (PI3K) play a key role in relating T‐cell activation to cytokine production 33. Immune cells uniquely express two of the four isoforms of type I PI3K: PI3K‐δ, engaged by receptor tyrosine kinases, such as those stimulated by T or B cell receptor ligation; and PI3K‐γ, usually activated by G‐protein coupled receptors such as chemokine receptors 34. These differences in signaling pathways predict that inhibitors that target PI3K‐δ, rather than PI3K‐γ, are more likely to reduce T‐cell cytokine responses.

To test the requirement for different PI3K isoforms for cytokine production, we added PI3K inhibitors that target either PI3K‐γ or PI3K‐δ to the restimulation coculture. Cytokine responses by primary responding and memory IAV‐specific CD4 and CD8 T cells from all organs were consistently reduced by the PI3K‐δ inhibitor (Supporting Information Fig. 7). In contrast, the PI3K‐γ inhibitor had little or no effect on cytokine production.

**Figure 4:**
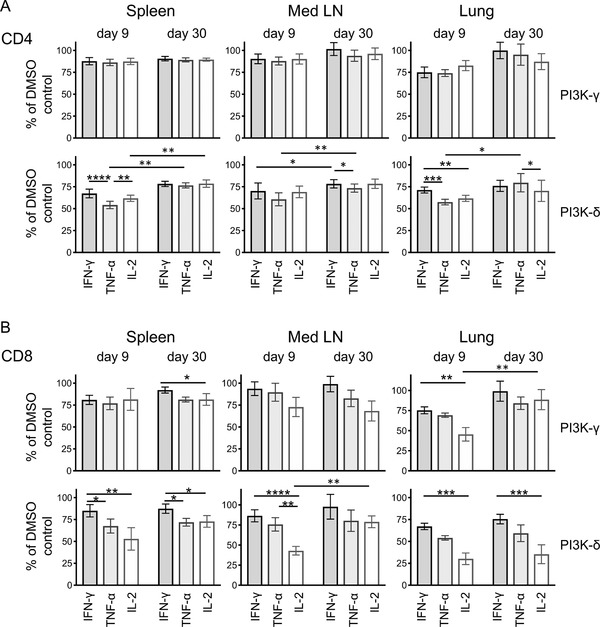
Memory CD4 T‐cell cytokine production is less affected by PI3K inhibitors than primary responding CD4 T cells. T cells from the spleen, Med LN, and lung were isolated from C57BL/6 mice infected with IAV either 9 or 30 days previously and reactivated with IAV+ bmDCs for 6 h in the presence of Golgi plug and the indicated PI3K inhibitor. The percentages of cytokine+ CD4 (A) and CD8 (B) T cells in each sample in comparison to that sample's DMSO control was calculated. Data are combined from three independent experiments at each time point with four to five samples per experiment per time point, expect that some samples were removed from the primary responses due to lack of IL2+ cells in the DMSO control: one sample removed from the CD4 T‐cell lung analysis; one sample removed from the CD8 Med LN analysis; and two from the lung analysis. Comparisons between cytokines within a time point were made using paired Friedman test with Dunn's multiple comparison; comparisons between time points were calculated using a Kruskal–Wallis with Dunn's multiple comparison test. *p < 0.05; **p < 0.01; ***p < 0.001; ****p < 0.0001.

To investigate whether the inhibitors affected the three different cytokines distinctly and whether these were different between primary and memory cells, we calculated the percentages of cytokine response for each organ from each sample to that in the relevant DMSO control (Fig. 4). For both CD4 and CD8 T cells, IFN‐γ tended to be least affected and TNF‐α most likely to be reduced for CD4 T cells and IL‐2 most reduced for CD8 T cells. Memory CD4 T cells from the spleen and lung were less sensitive to the inhibitors than primary T cells. This pattern was much less apparent for memory CD8 T cells with only IL‐2 responses in the lung and mediastinal lymph node less affected in the memory as compared to the primary response by the P3K‐γ and PI3K‐δ inhibitors, respectively.

**Figure 5:**
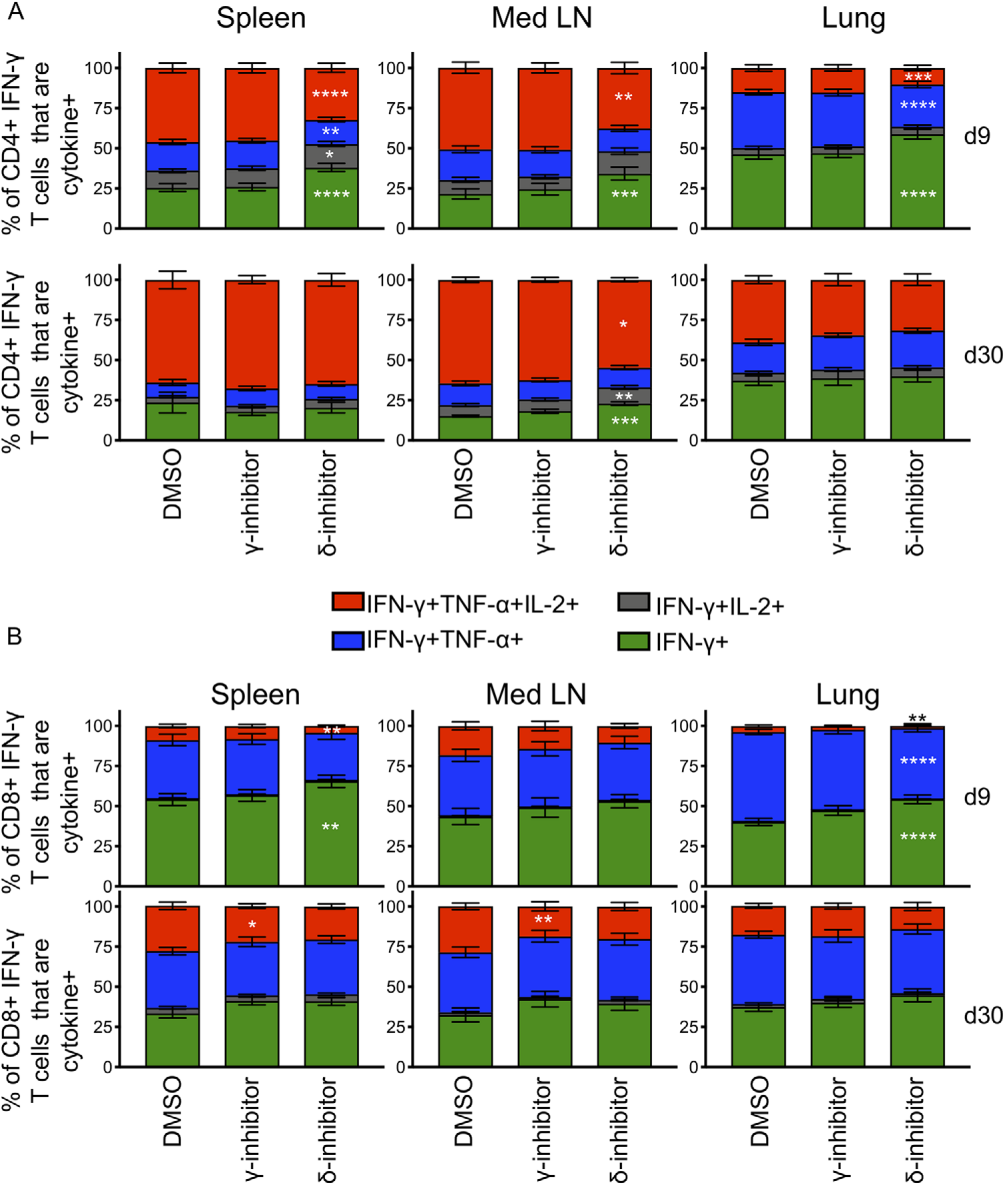
Multifunctional memory CD4 T cells are less affected by PI3K inhibitors than primary responding cells. T cells from the spleen, Med LN, and lung were isolated from C57BL/6 mice infected with IAV either 9 or 30 days previously and reactivated with IAV+ bmDCs for 6 h in the presence of Golgi plug and the indicated PI3K inhibitor. The percentages of IFN‐γ+ CD4 (A) and CD8 (B) T cells that also produced TNF‐α and/or IL‐2 were examined. Data are combined from three independent experiments at each time point with four to five samples per experiment per time point. Samples were analyzed using an ANOVA with Bonferroni's multiple comparison test comparing each inhibitor to the DMSO control. *p < 0.05; **p < 0.01; ***p < 0.001; ****p < 0.0001.

We also examined how the inhibitors affected the proportion of single, double, and triple cytokine producing T cells (Fig. 5). As IFN‐γ was least affected by the inhibitors, it was not surprising that the proportion of IFN‐γ single+ CD4 T cells increased in the presence of the PI3K‐δ inhibitor. This pattern was apparent in all organs in the primary response but only slightly altered in memory CD4 T cells from the mediastinal LN and not at all in memory CD4 T cells from the spleen and the lung.

In contrast, the effects of the inhibitors were less clearly distinct between primary and memory CD8 T cells. Triple cytokine producing cells were slightly reduced by the PI3K‐δ inhibitor in spleen and lung in primary CD8 T cells and not at all in memory CD8 T cells. Surprisingly, the PI3K‐γ inhibitor reduced the triple cytokine positive population in the memory T cells isolated from the spleen and mediastinal LN. Overall, these data again highlight the functional superiority of CD4, but not of CD8, T cell cytokine production in the memory as compared to the primary pool.

## Discussion

Our data demonstrate that memory cytokine producing IAV‐specific CD4 T cells have distinct characteristics from the population of activated CD4 T cells from which they are generated. Memory CD4 T cells were much more likely to demonstrate characteristics of multifunctional T cells, produced sustained cytokine responses, and were less reliant on signals mediated by PI3Kinase‐δ to produce cytokine. Interestingly, primary responding lung CD4 T cells did display a sustained cytokine response, suggesting that these cells may be regulated distinctly from those in lymphoid organs.

In contrast, while we confirm that CD8 memory T cells are more likely to produce IL‐2 11, 31, 32, their sensitivity to PI3K inhibitors was broadly similar to primary responding cells. Moreover, primary and memory CD8 T cells displayed similar kinetics in their cytokine secretion with a sustained or increasing response evident throughout the 6 h restimulation. Together, these data show that, based on these parameters, memory CD4, but not CD8 T cells display superior behaviors than primary responding cells.

In all organs, we found that T cells could produce effector cytokines, indicating that effector memory T cells were as likely to be found in lymphoid as nonlymphoid organs. The increased proportion of triple cytokine+ CD4 and CD8 T cells suggests that these cells may preferentially survive. Alternatively, these cells may mature into this phenotype following contraction, or have improved survival over time. This last hypothesis is supported by the continued increase in the proportion of multifunctional cells from day 30 to day 75 in some organs. It is experimentally difficult to discriminate between these possibilities. There are no surface markers that discriminate between the cytokine+ populations and neither cytokine secretion assays nor reporter mice can discriminate between single, double, and triple producers.

It is not clear why multifunctional T cells increase within the memory pool. Potentially, the production of IL‐2 provides autocrine survival signals 18. However, if autocrine IL‐2 production was sufficient, we would expect an increased predominance of IL‐2 single or double producing cells. This was not the case suggesting that other factors, potentially working with IL‐2, promote memory T‐cell generation and/or survival. Whether these factors are directly linked to the coproduction of these three cytokines or multifunctional cytokine production is merely a marker, is currently unclear. One potential explanation is that the repertoire of the memory pool is skewed toward specificities that are more likely to be multifunctional. However, there is little evidence for a loss of breadth within the IAV CD4 T cell response from the peak to day 60 postinfection 22.

Previous studies have compared the cytokine profiles of activated and memory CD4 T cells and found similar cytokine profiles between primary and memory populations 35, 36. In these cases, T cells in lymphoid organs were analyzed and only a single epitope was examined. This limits the breadth of these analyses potentially explaining differences with our data. Indeed, precursor frequency and epitope specificity are likely to influence cytokine response 19.

We believe analyzing a polyclonal antigen‐specific population, rather than a single epitope response, is an advantage. It is likely, however, that our assay does not identify all responding IAV‐specific T cells as some epitopes may not be generated by the bmDCs 37 and we are unable to identify IAV‐specific T cells that do not produce cytokine. Our data on CD8 T cells do, however, correlate with findings from studies using immunodominant IAV epitopes 11, 31, 32. A further caveat to our study is that the longest time period of in vitro reactivation examined was 6 h. It will be important to examine longer reactivation periods enabling a greater understanding of the difference between primary responding and memory T cells in terms of their ability to produce a sustained cytokine response.

The reduced sensitivity of memory CD4 T cells to the PI3K inhibitors and their more sustained cytokine response suggests that these cells are less reliant on strong activation signals than primary responding cells. This may be a consequence of increased expression of the key signaling molecule, Zap70 38, or altered association of signaling molecules within lipid rafts 39. Previous studies demonstrated reduced T cell activation and cytokine responses in either the absence of PI3Ks or in the presence of inhibitors 40, 41, 42, 43, 44. However, in these studies, the effect of the loss of PI3K signaling on cytokine production by CD4 T cells could not be uncoupled from initial effects on T cell priming. In contrast, we have directly demonstrated that inhibition of PI3K‐δ reduces TCR driven cytokine responses.

Currently, PI3K inhibitors are being tested in clinical trials for chronic inflammatory lung diseases including chronic obstructive pulmonary disease and asthma 45. IAV‐triggered exacerbations are a major cause of hospitalization in these patients 46. As IFN‐γ+ IAV‐specific memory CD4 and CD8 T cells have been associated with protection from disease following IAV infection in humans, it is unlikely that PI3K inhibitors will interfere with protective T cell memory 28, 47, 48, 49, 50. In contrast, primary virus‐specific immune responses may be more likely to be reduced by PI3K inhibitors and the cells most likely to be affected are those most associated with immune protection, multifunctional cytokine producing T cells. Choosing the most effective PI3K inhibitor that has the least impact on T cell cytokine production is an important clinical consideration. The PI3K‐γ inhibitor had only a limited effect on T cell cytokine responses suggesting it offers a good compromise.

In summary, our data demonstrate that the memory T cell pool is not simply a mirror image of the primary response. While the location of these two populations is similar, memory CD4 and CD8 T cells have a greater capacity to make broader cytokine responses. CD4 T cells were also altered in their sensitivity to TCR signaling inhibitors and produced a more sustained cytokine response, suggesting functional maturation as these cells develop into memory cells. These novel findings pave the way for an improved understanding of the signals that regulate memory CD4 T cell generation.

## Materials and methods

### Animals and infections

Ten week‐old female C57BL/6 mice were purchased from Envigo (http://Huntingdon,UK). They were maintained at the University of Glasgow under standard animal husbandry conditions in accordance with UK home office regulations (Project License P2F28B003) and approved by the local ethics committee. Following 1 week of acclimatization, the mice were briefly anesthetized using inhaled isoflurane and infected with 200–300 plaque forming units of IAV strain WSN in 20 µL of PBS intranasally. IAV was prepared and tittered in MDCK cells. Infected mice were weighed daily. Any animals that lost more than 20% of their starting weight were removed from the study and humanely euthanized. Group sizes were based on previous experiments considering the known variability of the antiviral T‐cell response, animals that did not lose any weight following infection were excluded.

### Tissue preparation

Where indicated, mice were injected i.v. with 1 µg of anti‐CD45 (30‐F11) labelled with Alexa 488 (eBioscience, Santa Clara, CA) and euthanized by cervical dislocation 3 min later. Alternatively, mice were euthanized with a rising concentration of carbon dioxide and perfused with PBS‐5 mM EDTA to remove blood cells from the lungs. Spleen and mediastinal lymph nodes were processed by mechanical disruption. Single cell suspensions of lungs were prepared by digestion with 1 mg/mL collagenase and DNAse (Sigma, St. Louis, MI) for 40 min at 37°C. At day 30, too few cells were recovered from the MLN for the analysis of PI3K inhibitor sensitivity. Therefore, each of the organs from two mice that had lost similar amounts of weight were combined to provide 1 day 30 sample. In the PI3K inhibitor experiments, T cells were isolated using Stemcell mouse T‐cell isolation kits following the manufacturer's recommendations.

### Influenza virus and control antigen

The IAV antigen was prepared using a similar protocol as descried 51. Briefly, T75 flasks of 80% confluent MDCK cells were incubated for 1 h at 37°C, 5% CO_2_ with or without an MOI of 0.001 IAV WSN. Virus or control inoculums were removed and cells incubated for a further 2 days in 12 mL of OPTI‐MEM supplemented with Pen/Strep and 1.0 µg/mL trypsin‐TPCK. After 48 h, the cells were harvested, spun down, resuspended in 0.1 M glycine buffer containing 0.9% NaCl, pH9.75 and shaken at 4°C for 20 min, incubated in a sonication bath for 10 s intervals four times before centrifugation at 2000 rpm for 20 min at 4°C. Supernatant was aliquoted and frozen at −80°C.

### Bone marrow DCs

BmDCs were prepared as described 52. Briefly, bone marrow cells were flushed from the tibias and femurs of female C57BL/6 mice and RBCs removed. Cells were cultured in complete RPMI (RPMI with 10% fetal calf serum, 100 µg/mL penicillin‐streptomycin, and 2 mM l‐glutamine) at 37°C 5% CO_2_ in the presence of GM‐CSF (prepared from X‐63 supernatant 53) with media supplemented on day 2 and replaced on day 5. On day 7, DCs were harvested, incubated overnight with either control antigen or IAV antigen (MOI of 0.3).

### Ex vivo restimulation

Single cell suspensions were cocultured with bmDCs in complete RMPI at a ratio of approximately 10 T cells to 1 DC in the presence of Golgi Plug (BD Bioscience, Franklin Lakes, NJ). Cocultures were incubated at 37°C, 5% CO_2_ for 6 h unless stated. PI3K inhibitors were used at: PI3K‐δ, 100 nM; PI3K‐γ, 300 nM. These concentrations were selected as they are midrange for the concentrations known to affect T‐cell responses. All inhibitors were 300–1000‐fold selective over other PI3K family members.

### Flow cytometry

Cells were harvested and following incubation with Fc block (homemade containing 24G2 supernatant and mouse serum) surface stained with anti‐CD4 APC‐Alexa 780 (eBioscience; clone: RM4‐5), anti‐CD44 PerCP‐Cy5.5 (eBioscience; clone: IM7), CD8 PeCy7 (eBioscience; clone: 53–6.7), and “dump” antibodies: B220 (clone: RA3‐6B2) and MHC II (clone: M5114) both on eFluor‐450 (eBioscience) for 20 min at 4°C. Cells were stained with a fixable viability dye eFluor 506 (eBioscience) as per the manufacturer's recommendations. Cells were fixed with cytofix/cytoperm (BD Bioscience) for 20 min at 4°C and stained in permwash buffer with anticytokine antibodies for 1 h at room temperature (anti‐IFN‐γ PE (clone: XMG1.2;), anti‐TNF Alexa‐Fluor‐488 (clone: MP6‐XT22), anti‐IL‐2 APC (clone: JES6‐5H4) all from eBioscience. Following washing with permwash buffer, samples were acquired on a BD LSR or Fortessa and analyzed using FlowJo (version 10 Treestar). Data are presented as required for MIFlowCyt.

## Statistical analysis

Data were analyzed using Prism version 7 software (GraphPad). Differences between groups were analyzed by paired or unpaired ANOVAs as indicated in figure legends. In all figures * represents a *p* value of <0.05; **: *p* < 0.01, ***: *p* < 0.001, ****: *p* < 0.0001.

## Author contribution

LMW designed and performed experiments, analyzed data and edited the manuscript. KM, LM, JIG, and AF performed experiments; MT provided reagents and edited the manuscript; CSG designed research and edited the manuscript, MKLM designed and performed the research, analyzed data and wrote the manuscript.

## Conflict of Interest

MT was an employee of AstraZeneca during the study. CSG has received consulting fees from AstraZeneca (more than $10,000). PI3K inhibitor tools were generated as part of drug discovery projects aimed at modulating pulmonary immune responses. Any subsequent commercial interests had no influence on the design, performance, or interpretation of any experiment presented in this body of work.

**Abbreviations**
bmDCs: bone marrow‐derived dendritic cells
IAV: influenza A virus
PI3K: PI3Kinases

## Supporting information

Supporting Information
