# Supplementary material for: Multifunctional cytokine production reveals functional superiority of memory CD4 T cells
Source: Eur J Immunol. 2019 Jun 13;49(11):2019–29. doi: 10.1002/eji.201848026 (PMC6900100; doi:10.1002/eji.201848026)
Supplement: Supplementary file 1 — Supporting Information [file EJI-49-2019-s001.pdf]

European Journal of Immunology

Supporting Information
for
DOI 10.1002/eji.201848026

Lotus M Westerhof, Kris McGuire, Lindsay MacLellan, Ashley Flynn, Joshua I Gray, Matthew Thomas, Carl S Goodyear and Megan KL MacLeod

Multifunctional cytokine production reveals functional superiority of memory CD4 T cells

© 2019 The Authors. *European Journal of Immunology* published by WILEY-VCH Verlag GmbH & Co. KGaA, Weinheim.

Multifunctional cytokine production reveals functional superiority of memory CD4 T cells

Lotus M Westerhof[1,2], Kris McGuire[2$], Lindsay MacLellan[2], Ashley Flynn[1], Joshua Gray[1], Matthew Thomas[3]†, Carl S Goodyear[1,2], Megan KL MacLeod[1*]

Supplementary Information

Supplementary Figure 1

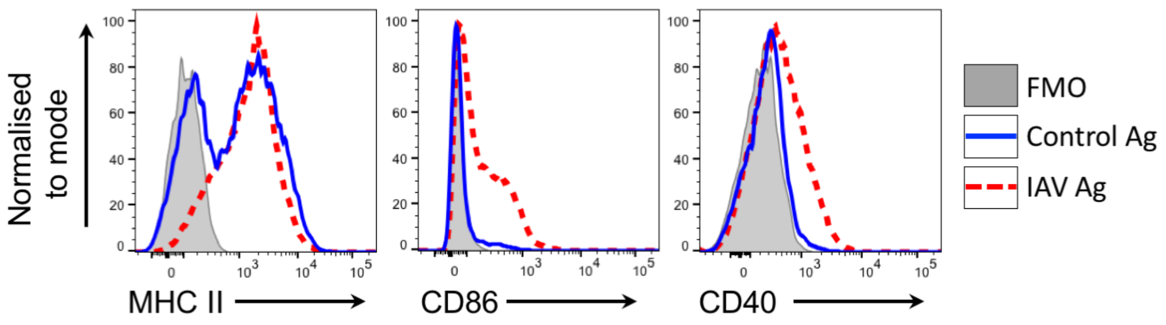

SF1: IAV antigen causes some upregulation of MHC II and costimulatory molecule expression on bmDCs

Bone marrow derived DC were cultured overnight with sonicated IAV infected (red dotted line) or uninfected MDCK cells (blue line). The levels of MHC II, CD86 and CD40 were examined on CD11c+ cells with Frequency Minus One (FMO)s shown in grey. Data are representative of 3 experiments.

Supplementary Figure 2

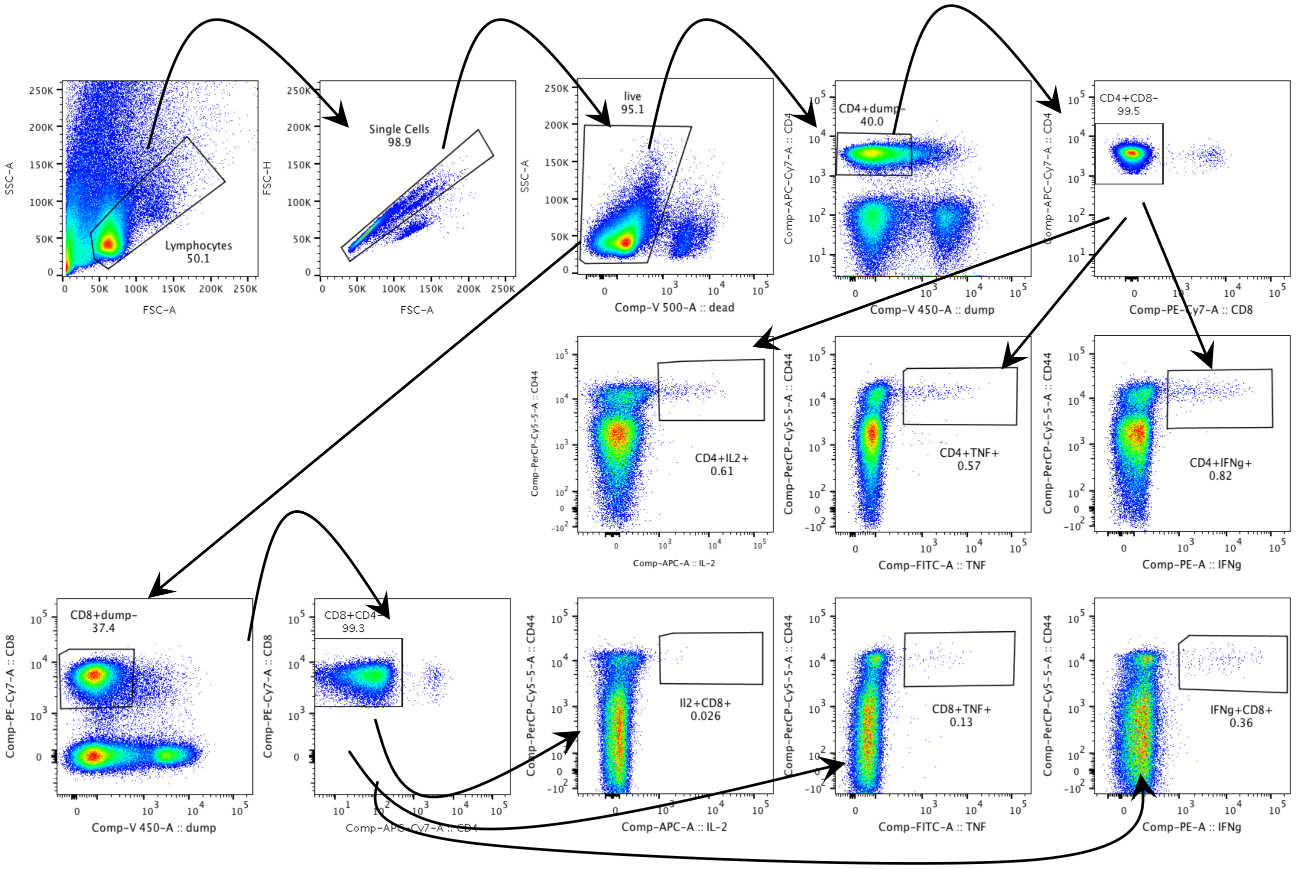

SF2: Gating scheme for T cell cytokine analysis. Cells are first gated through a lymphocyte gate, then on single cells and live cells. CD4 or CD8 T cells that do not express B220, or MHC II are then gated on before exclusion of cells that are CD8 or CD4 positive respectively. Cytokine positive cells are examined by plotting the cytokine versus the activation marker, CD44.

Supplementary Figure 3

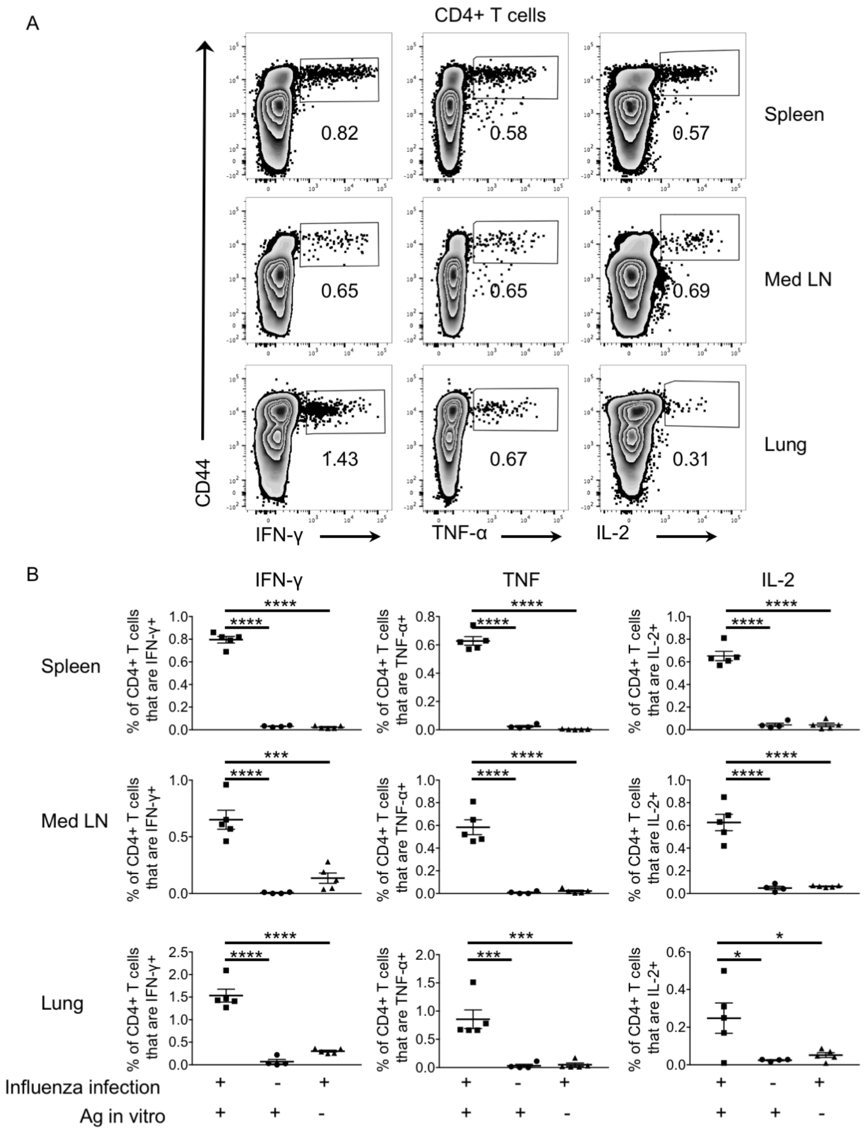

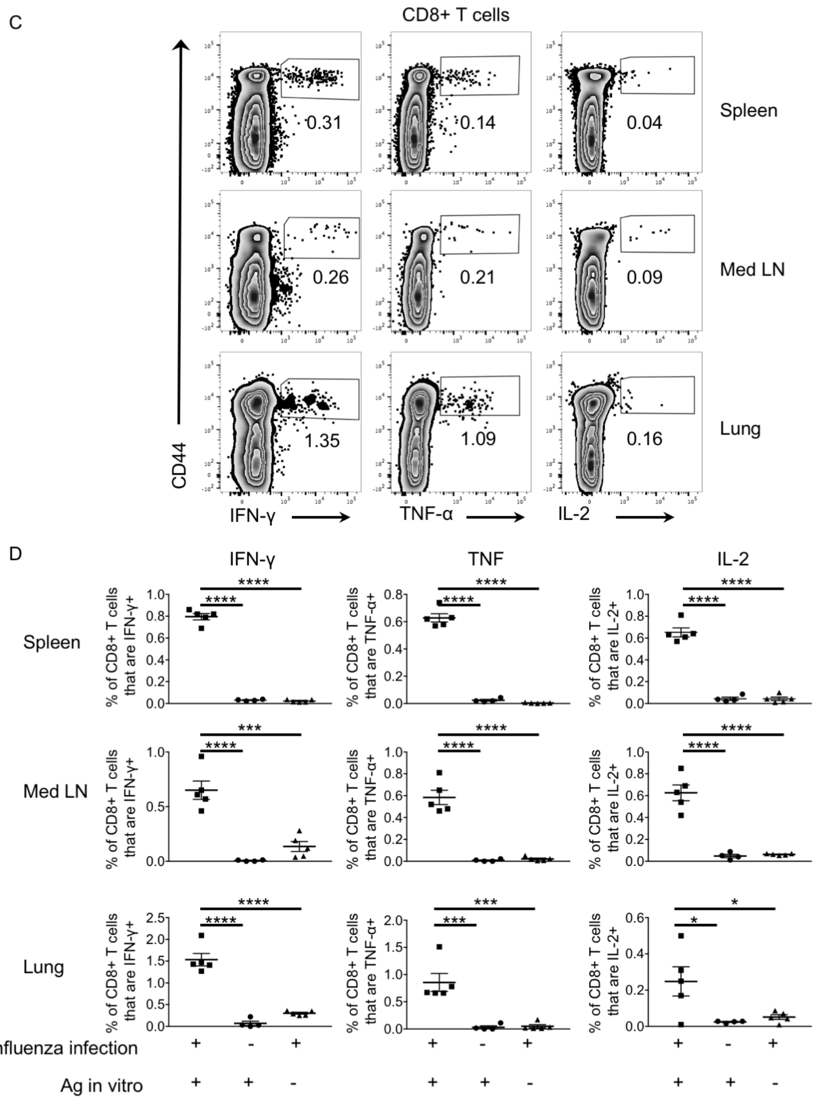

SF3: Identification of influenza virus specific cytokine producing CD4 and CD8 T cells

C57BL/6 mice were infected with 200-300PFU of influenza virus (WSN) intranasally. 9 days later, the percentages of IFN-γ, TNF-α, and IL-2 producing CD4 T cells (A, B) or CD8 T cells (C, D) were analysed following a 6 hour co-culture of T cells isolated from the spleen, lymph nodes or lungs of the infected mice with IAV+ bmDCs. Data are representative of three experiments with 4-6 samples per timepoint. Cells are gated on live dump negative CD4+ or CD8+ T cells and the numbers show the percentages of cytokine positive cells in the indicated gates.

Supplementary Figure 4

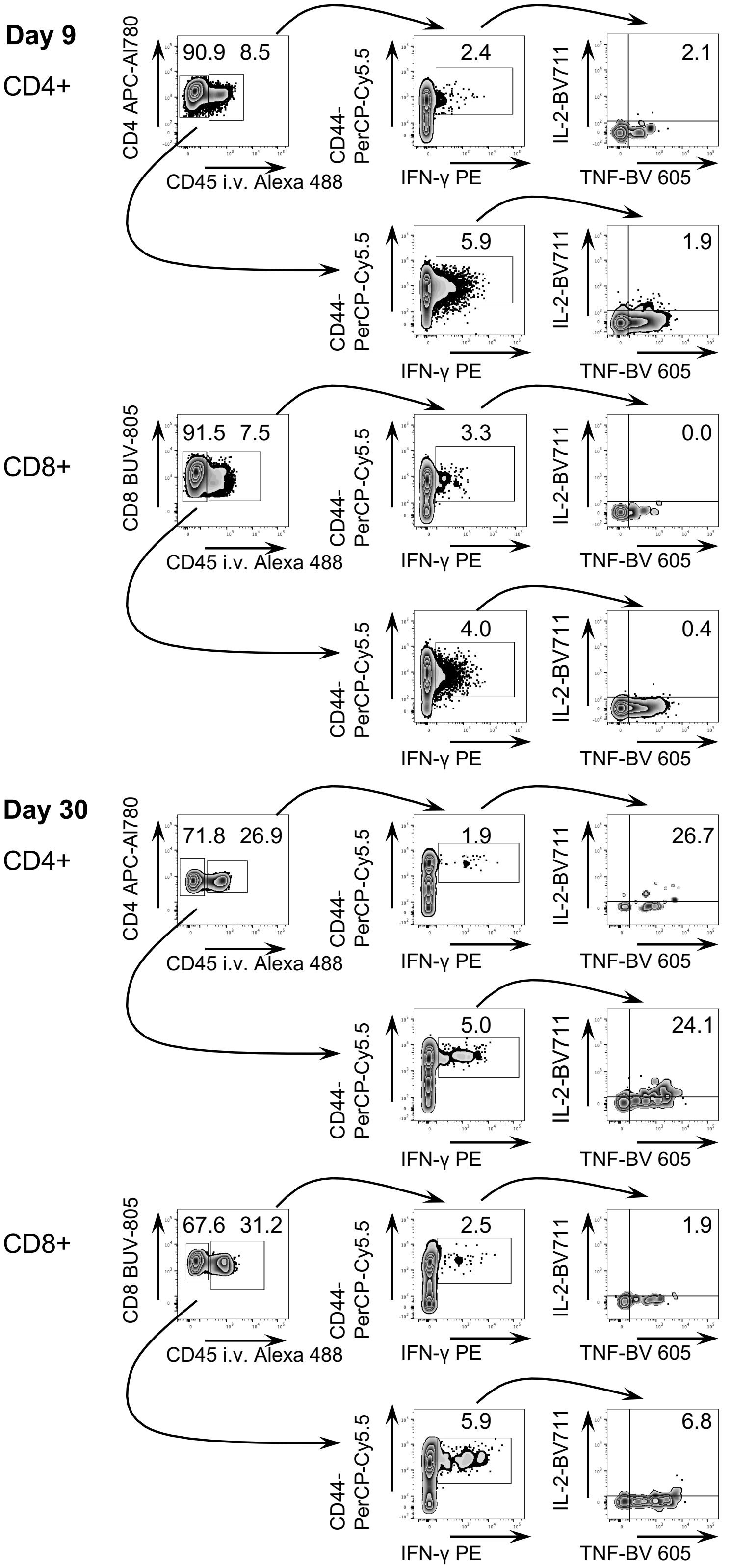

SF4: the proportion of IAV specific T cells that are present in lung vasculature increases from day 9 to day 30. C57BL/6 mice were infected with IAV and 9 or 30 days later fluorescently labelled anti-CD45 was injected i.v. 3 minutes before tissues were harvested and single cell suspensions from the lung co-cultured with IAV+ bmDCs for 6 hours. Cells are gated as in SF2 and on CD4 or CD8 cells as indicated and then through subsequent gates as shown on either CD45+/- cells to determine the proportions of these cells that were IFN-γ+, and the proportions of IFN-γ+cells that also expressed IL-2 and TNF-α. The numbers on the FACS plots show the percentages of cells in the indicated gates or quadrants. Data are from 3 experiments with 3-4 mice per group.

Supplementary Figure 5

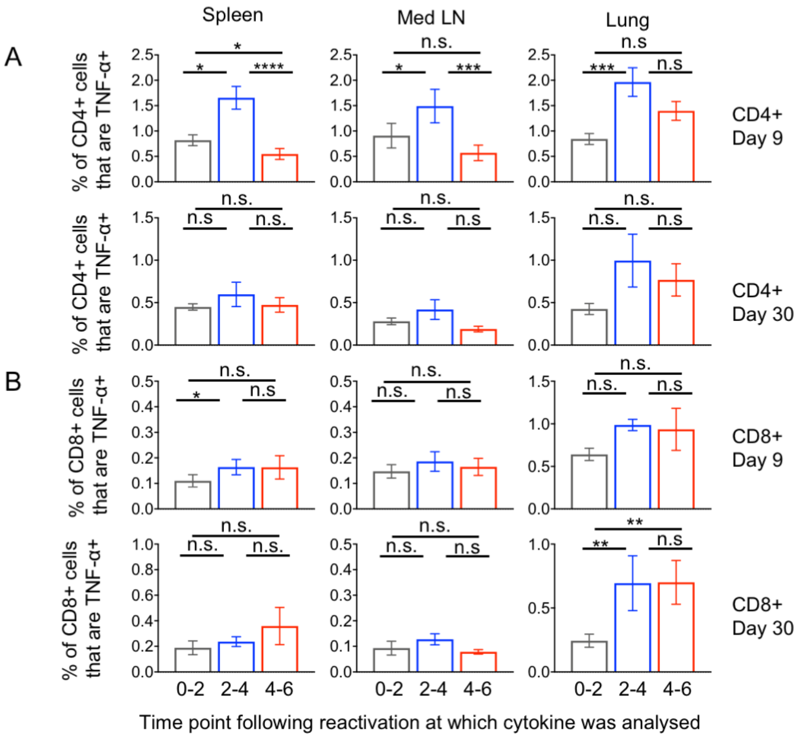

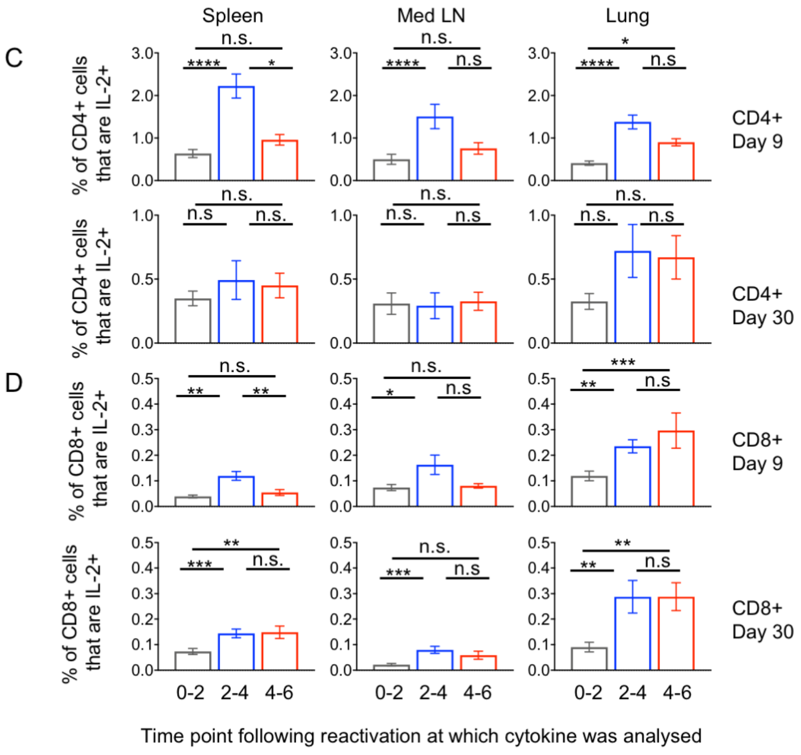

SF5: Memory CD4 T cells demonstrate more sustained cytokine production than primary responding cells

Mediastinal lymph nodes, spleens and lungs were taken from C57BL/6 mice 9 and 30 days post-infection with IAV and reactivated in vitro with IAV+ bmDCs for 2, 4 or 6 hours in the presence of Golgi plug for the last 2 hours of culture. The percentages of TNF-α+ (A,B) and IL-2+ (C,D) CD4 (A, C) and CD8 T cells (B, D) were examined at the indicated time points. Error bars show SEM. Data are combined from 3 experiments per timepoint with 4 mice per timepoint in each experiment. Samples were analysed using a Friedman's paired test followed by a Dunn's multiple comparison test *: $p<0.05$; **$p<0.01$; ***$p<0.001$; ****$p<0.0001$.

Supplementary Figure 6

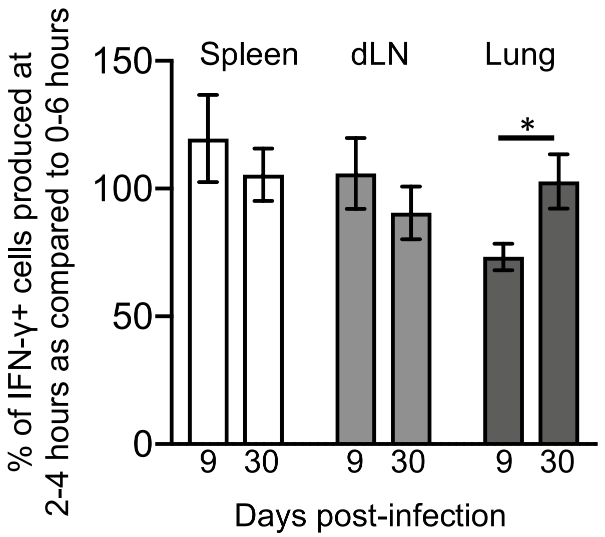

Supplementary Figure 6: Similar proportions of CD4 T cells producing IFN-γ at 2-4 hours are present in the primary responding and memory T cell pools

Mediastinal lymph nodes, spleens and lungs were taken from C57BL/6 mice 9 and 30 days post-infection with IAV and reactivated in vitro with IAV+ bmDCs for 4 or 6 hours with Golgi plug present either at 2-4 hours or the whole culture respectively. The numbers of IFN-γ+ CD4 T cells present were calculated from the ex vivo counts and the flow cytometry data. The graph shows the percentages of CD4+ IFN-γ+ cells present at 2-4hours as compared to 0-6hours. Error bars show SEM. Data are combined from 3 experiments per timepoint with 4 mice per timepoint in each experiment. Samples were analysed using an unpaired t-test*: $p<0.05$.

Supplementary Figure 7

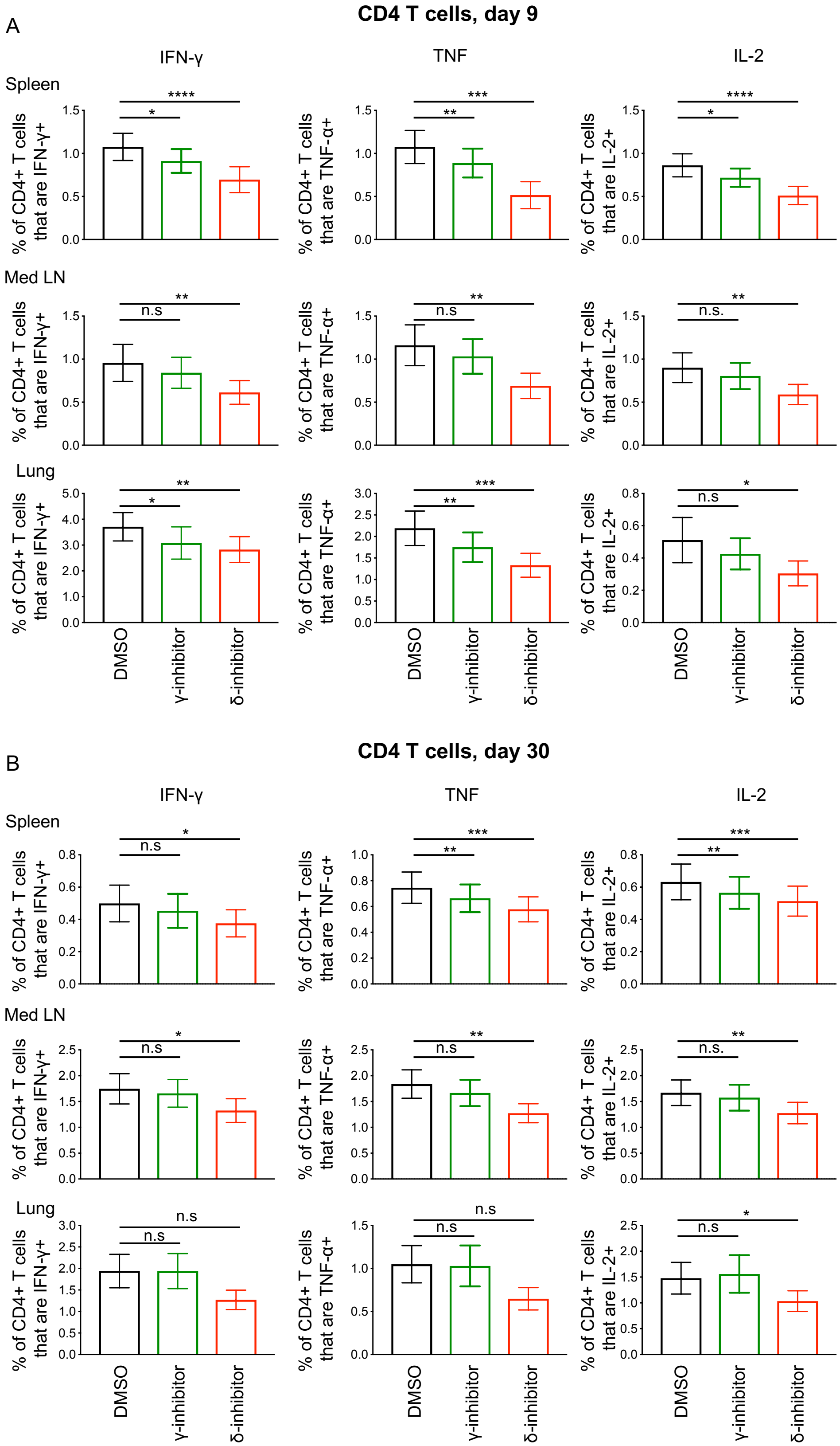

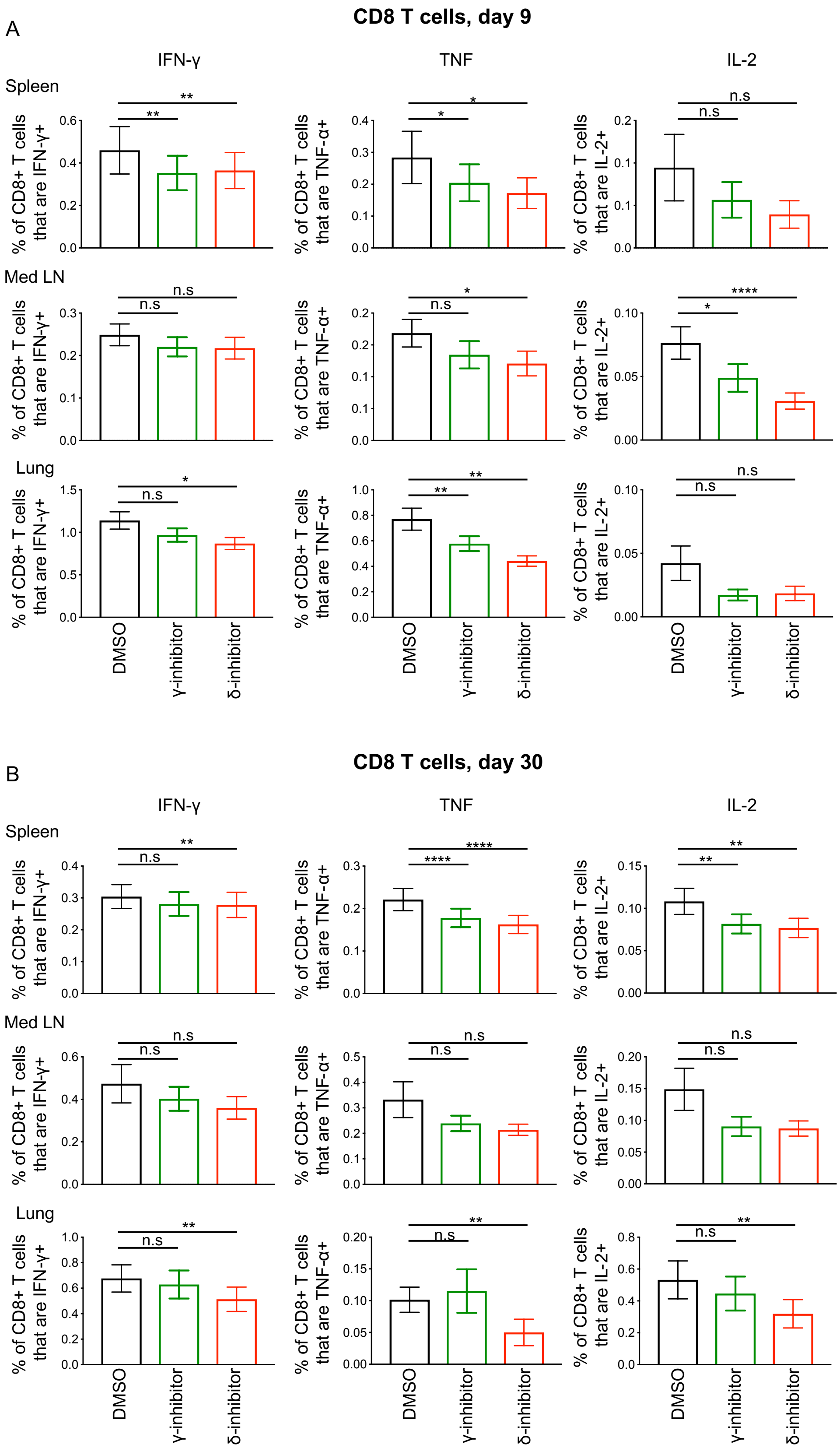
A
CD8 T cells, day 9
IFN-γ
TNF
IL-2
Spleen
Med LN
Lung
% of CD8+ T cells that are IFN-γ+
% of CD8+ T cells that are TNF-α+
% of CD8+ T cells that are IL-2+
DMSO
γ-inhibitor
δ-inhibitor
B
CD8 T cells, day 30

SF7: PI3Kinase delta inhibitor reduces the proportion of cytokine producing T cells

C57BL/6 mice were infected with IAV and 9 (A, C) or 30 (B, D) days later the percentages of IFN-γ, TNF-α, and IL-2 producing CD4 T cells (A, B) or CD8 T cells (C, D) were analysed following a 6 hour co-culture in the presence of the indicated PI3Kinase inhibitors of T cells isolated from the spleen, lymph nodes or lungs of the infected mice with bmDCs cultured overnight with sonicated influenza antigen. The data are combined from 3 independent experiments per time point with 4-5 samples per timepoint per experiment. Error bars are SEM and samples were analysed using paired ANOVA with Dunnett's multiple comparison test *: $p<0.05$; **$p<0.01$; ***$p<0.0001$; ****$p<0.0001$.
